# Assessment of the causal association between obstructive sleep apnea and telomere length: a bidirectional mendelian randomization study

**DOI:** 10.3389/fgene.2025.1294105

**Published:** 2025-03-04

**Authors:** Rongfang Xie, Shiyu Chen, Xiaojian Li, Zhihui Lan

**Affiliations:** ^1^ Department of Postgraduate, Jiangxi University of Chinese Medicine, Nanchang, Jiangxi, China; ^2^ Department of Respiration, Affiliated Hospital of Jiangxi University of Traditional Chinese Medicine, Nanchang, Jiangxi, China

**Keywords:** obstructive sleep apnea, telomere length, causality, genetic association, mendelian randomization

## Abstract

**Background:**

A plethora of observational studies has established a significant correlation between Obstructive Sleep Apnea (OSA) and Telomere Length (TL). Nevertheless, a universal consensus on precise causal association and its directionality has not yet been achieved. To shed light on this, we employed Mendelian Randomization (MR) to investigate the bidirectional causal association between OSA and TL.

**Method:**

Utilizing publicly accessible Genome-Wide Association Studies (GWAS) datasets, we procured genetic data pertinent to MR analysis. The study incorporated samples from both the OSA (n = 217,955) and TL (n = 472,174) cohorts. In the forward MR analysis, OSA served as the exposure variable and TL as the outcome. Conversely, the reverse MR analysis treated TL as the exposure and OSA as the outcome. We employed the Inverse variance weighted (IVW) as the primary methodology for MR analysis. To ensure the robustness of our MR findings, multiple sensitivity analyses were performed.

**Results:**

In the forward MR analysis, a negative correlation was indicated between OSA and TL (IVW: odds ratio (OR) = 0.964, 95% confidence interval (CI): 0.939–0.980, P = 0.006 < 0.05). However, no significant association was identified between TL and the risk of OSA in the reverse MR analysis (IVW: OR = 0.965, 95% CI: 0.870–1.070, P = 0.499 > 0.05).

**Conclusion:**

Our study indicated a potential association between OSA and the increased risk of shorter TL, offering vital academic support for future clinical studies on this association.

## 1 Introduction

OSA is a sleep-associated respiratory disorder (Schütz et al., 2021). Its prevalence has surged recently (Peppard et al., 2013; Ghavami et al., 2023), affecting approximately 100 million adults worldwide (Benjafield et al., 2019). OSA primarily arises from the obstruction of the upper respiratory pathway, which restricts airflow. Those afflicted with OSA often experience apnea or hypoventilation during sleep, leading to consistent intermittent hypoxia and repeated sleep interruptions (Wallace et al., 2022). This sequence of pathophysiological reactions can induce chronic inflammation and oxidative stress, potentially resulting in cellular damage and early cellular aging (Gaspar et al., 2017; Turkiewicz et al., 2021). Persistent exposure to these conditions might compromise the integrity of various physiological systems, and in severe instances, pose mortal threats (Kapur et al., 2017; Bandi et al., 2021). Therefore, comprehensive research into OSA’s implications for human health is of paramount importance.

Telomeres, situated at the termini of eukaryotic chromosomes, are specific structures whose length is commonly assessed in white blood cells (O'Sullivan and Karlseder, 2010). Their fundamental role is to maintain chromosome integrity and stability. Owing to intrinsic constraints of cellular replication, telomeres aren't comprehensively replicated with each cellular division, resulting in their consistent attrition. Upon reaching a specific diminutive length, cells might suspend division, potentially precipitating premature senescence or apoptosis (Blackburn et al., 2015; Wang et al., 2018). Consequently, telomeres are recognized as pivotal biological markers of cellular aging, often termed the aging “timer” (Fumagalli et al., 2012). Existing research underscores the profound correlation between telomere attrition and elevated disease incidence and mortality (Aung et al., 2023; Wang et al., 2023; Zhu S. et al., 2023). Thus, preserving telomere stability is imperative for disease prevention and the deceleration of cellular aging.

In recent years, the relationship between OSA and TL has garnered considerable attention, particularly the contentious debate over OSA as a potential risk factor for telomere shortening (Turkiewicz et al., 2021). The majority of studies highlighted a negative correlation between OSA severity and TL (Barceló et al., 2010; Bhatt et al., 2021; Pinilla et al., 2021). Contrarily, a few studies suggested that children diagnosed with OSA might experience telomere elongation instead of reduction (Kim et al., 2010). Another study proposed a J-shaped relationship between TL and the severity of OSA, suggesting that patients with moderate to severe OSA might have longer telomeres (Polonis et al., 2017). Interestingly, research indicated shorter TL in high-risk female OSA patients, and this trend was independent of income, age, obesity, smoking, hypertension, alcohol consumption, and education level, but such a trend was absent in their male OSA counterparts (Riestra et al., 2017). In contrast, another study emphasized OSA as a significant factor leading to telomere shortening in middle-aged men (Boyer et al., 2016). Clarifying the exact relationship between OSA and TL is crucial for a deeper understanding of OSA and its impact on human health. However, given the conflicting findings on TL variations in OSA patients and the absence of definitive evidence on whether TL changes occur before or after OSA onset, discerning a clear causal relationship remains elusive.

MR analysis is an analytical approach that leverages genetic variations (single nucleotide polymorphisms, SNP) as instrumental variables (IVs) to infer the causal relationship between exposure and outcome (Xu et al., 2022). Given its capacity to circumvent confounding factors and reverse causality, the findings derived from MR are deemed more credible. In this study, we employed a bidirectional two-sample MR strategy to probe the potential causal association between OSA and TL.

## 2 Methods

### 2.1 Research design

In alignment with the guidelines of the STROBE checklist for MR studies (STROBE-MR), we undertook a bidirectional MR analysis to assess the bidirectional association between OSA and TL (Skrivankova et al., 2021). For the validity of this analysis, it was essential that three core assumptions be met (Davies et al., 2018): ① IVs must be strongly associated with the exposure; ② IVs must be independent of any confounders that could affect the outcome; ③ IVs affect the outcome only through their association with the exposure, and not through any other pathways.

### 2.2 Data source

For this study, we utilized summary data from two GWAS (https://gwas.mrcieu.ac.uk/) of European ancestry. Genetic information for OSA was sourced from the publicly available GWAS data in the FinnGen database, featuring 16,761 cases and 201,194 controls (Table 1). The diagnosis of OSA relied on ICD codes (ICD-10: G47.3; ICD-9: 3472A), which were obtained from the Finnish National Hospital Discharge Registry and the Cause of Death Registry. This diagnosis was based on subjective symptoms, clinical examination, and sleep registration, with a threshold of AHI ≥5 events·h^-1^ or a respiratory event index ≥5 events·h^-1^ serving as key indicators for confirmation (Strausz et al., 2021). The GWAS data for TL was derived from the United Kingdom Biobank (UKB), comprising 472,174 adults with specific traits (Codd et al., 2021) (Table 1). This research strictly relied on samples of European ancestry, effectively eliminating confounding factors associated with racial variations. As the data we employed are publicly available, no supplementary ethical approval was required.

**TABLE 1:** Summary of the GWAS.

Trait	GWAS ID	Sample size	N SNPs	Population	PubMed ID
OSA	finn-b-G6_SLEEPAPNO	217,955	16,380,465	European	33,243,845
TL	ieu-b-4879	472,174	20,134,421	European	34,611,362

OSA, obstructive sleep apnea; TL, telomere length; N SNPs, Numbers of single nucleotide polymorphisms.

### 2.3 Selection of IVs

In accordance with the objective of identifying SNPs significantly correlated with exposure, we adopted a genome-wide significance threshold of P < 5 × 10^−8^. However, under this criterion, many SNPs related to OSA were not identified. Thus, we adjusted our criterion to a more lenient P < 5 × 10^−7^ for isolating OSA-associated SNPs (Zhu Q. et al., 2023). Employing the PLINK clustering technique, we excluded SNPs in linkage disequilibrium (r2 > 0.001; aggregation window: 10,000 KB), retaining only the SNPs with the most significant P-values. During data integration, we discarded palindromic SNPs. To ascertain potential biases within weak IVs, the strength of IVs was assessed using the F-statistic. Previous research indicated that an F-statistic exceeding 10 signifies a reduced likelihood of IV bias (Zhu Q. et al., 2023).

### 2.4 Statistical analysis

In version 4.3.0 of R software, the “TwoSampleMR” package was utilized for causality assessment. Four MR methods were selected for analysis: IVW, Weighted Median (WM), weighted mode, and MR-Egger regression. IVW evaluates the variance of each SNP’s effect estimation and assigns more weight to the SNPs considered to be more stable and precise, enhancing the statistical robustness of the overall effect estimation (Burgess et al., 2013). Thus, we primarily adopted IVW for our MR analysis. However, caution is required as associations between certain genetic variants and non-exposed confounders may introduce bias into IVW results (Bowden et al., 2017). To ensure the stability of our results, we also implemented MR-Egger regression, WM, and weighted mode methodologies. MR-Egger regression can adjust for potential confounders and is somewhat tolerant to weaker IVs, but it generally requires a large sample size (Burgess and Thompson, 2017). WM yields consistent estimates even when up to 50% of the genetic variations prove ineffective (Bowden et al., 2016). Additionally, the weighted mode has a relatively lenient assumption regarding the efficacy of genetic variations.

In MR analysis, when a specific SNP affects the outcome but this influence is independent of the exposure’s causal relationship, it’s termed horizontal pleiotropy. This phenomenon could introduce biases in MR results. To comprehensively assess potential horizontal pleiotropy, we employed several analytical methodologies: Firstly, we used MR- Pleiotropy RESidual Sum and Outlier (MR-PRESSO) to detect outliers that might violate the causal effect (Ong and MacGregor, 2019). Subsequently, MR-Egger regression was conducted. A substantial deviation of its intercept from zero indicates horizontal pleiotropy. Lastly, by adopting a“leave-one-out” study, we systematically eliminated each SNP to compare the MR findings of the residual SNPs with the aggregate MR results. Furthermore, given the potential varying impact of distinct SNPs on exposure, such variations can lead to heterogeneity. Thus, Cochrane’s Q value was utilized to gauge the heterogeneity among SNPs.

## 3 Results

### 3.1 Forward MR analysis: causality of OSA on TL

**FIGURE 1:**
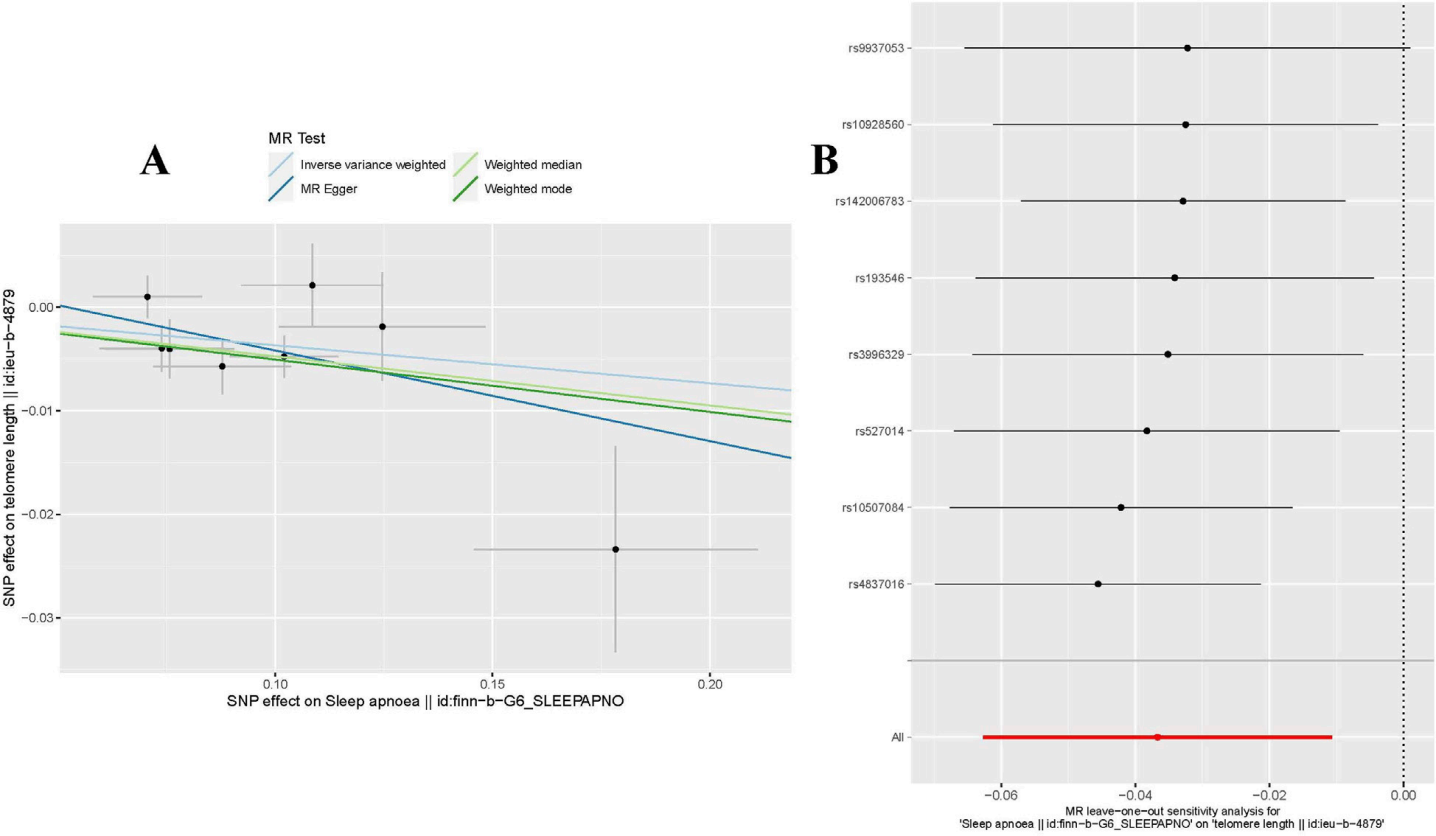
MR analysis for OSA on TL. **(A)** The scatter plot illustrated an intuitive depiction of the relationship between OSA-related IVs and TL. **(B)** The “Leave-one-out” sensitivity analysis enables the identification of bias-inducing SNPs, further elucidating their potential impact on the overall causal estimation. OSA, Obstructive Sleep Apnea; TL, Telomere Length; instrumental variables, IVs; SNPs, single nucleotide polymorphisms.

In our forward MR analysis, we adopted 8 SNPs linked with OSA (P < 5.00 × 10^−7^) to explore their latent effects on TL. The F-statistic for each SNP exceeded 10, with values ranging from 26.162 to 66.586. Details were provided in Supplementary Material: Supplementary Table S1. Based on the IVW method, we found a significant negative causal relationship between OSA and TL (OR = 0.964, 95%CI: 0.939–0.989, P = 0.006 < 0.05, Table 2). This relationship was reinforced by results from both WM (OR = 0.954, 95%CI: 0.926–0.983, P = 0.002 < 0.05, Table 2) Weighted Mode (OR = 0.951, 95%CI: 0.914–0.989, P = 0.032 < 0.05, Table 2). Yet, findings from the MR-Egger regression did not confirm a distinct causal association between OSA and TL (OR = 0.916, 95%CI: 0.802–1.050, P = 0.246 > 0.05, Table 2). We have visualized these causal estimates in a scatter plot (Figure 1A).

**TABLE 2:** Forward MR analysis of OSA on TL.

MR methods	N SNPs	β	SE	OR (95%CI)	p-value
IVW	8	−0.037	0.013	0.964 (0.939–0.989)	0.006
MR Egger	8	−0.087	0.068	0.916 (0.802–1.050)	0.246
Weighted median	8	−0.047	0.015	0.954 (0.926–0.983)	0.002
Weighted mode	8	−0.050	0.019	0.951 (0.914–0.989)	0.032

OSA, obstructive sleep apnea; TL, telomere length; N SNPs, Numbers of single nucleotide polymorphisms; MR, mendelian randomization; SE, standard error; β, causal effect coefficient; OR, odds ratio; IVW, inverse variance weighted.

To evaluate the stability of the aforementioned findings, we conducted sensitivity analyses and heterogeneity tests. The MR-PRESSO test revealed no outliers that could disrupt the causal relationship (P = 0.241 > 0.05). The MR-Egger regression further confirmed that the study results were uninfluenced by horizontal pleiotropy (P = 0.475 > 0.05, Table 3). Using the “Leave-one-out” technique for sensitivity analysis revealed that the stepwise exclusion of individual SNPs exerted no substantial influence on the causal relationship estimates (Figure 1B). Additionally, Cochran’s Q test demonstrated no evidence of heterogeneity in either IVW (P = 0.178 > 0.05, Table 3) or MR-Egger regression (P = 0.158 > 0.05, Table 3).

**TABLE 3:** Analysis of heterogeneity and pleiotropy in forward MR.

MR methods	p-value for heterogeneity	egger_intercept	p-value for pleiotropy
IVW	0.178		
MR Egger	0.158	0.005	0.475

MR, mendelian randomization; IVW, inverse variance weighted.

### 3.2 Reverse MR analysis: causality of TL on OSA

In reverse MR analysis, SNPs associated with TL were employed as the IVs to evaluate their influence on OSA. After data consolidation, four palindromic SNPs (rs2276182, rs2306646, rs56178008, and rs670180) were excluded, and 130 validated SNPs were chosen as IVs (P < 5.00 × 10^−8^). See Supplementary Material for specifics: Supplementary Table S2. According to the IVW results, there was no significant causal relationship between TL and OSA. This finding was corroborated by other MR analyses (Table 4). Moreover, no horizontal pleiotropy or heterogeneity was observed (Table 5).

**TABLE 4:** Reverse MR analysis of TL on OSA.

MR methods	N SNPs	β	SE	OR (95%CI)	p-value
IVW	130	−0.036	0.053	0.965 (0.870–1.070)	0.499
MR Egger	130	−0.115	0.0891	0.890 (0.742–1.070)	0.219
Weighted median	130	−0.068	0.083	0.934 (0.797–1.095)	0.412
Weighted mode	130	−0.119	0.095	0.888 (0.743–1.061)	0.211

OSA, obstructive sleep apnea; TL, telomere length; N SNPs, Numbers of single nucleotide polymorphisms; MR, mendelian randomization; SE, standard error; β: causal effect coefficient; OR, odds ratio; IVW, inverse variance weighted.

**TABLE 5:** Analysis of heterogeneity and pleiotropy in reverse MR.

MR methods	p-value for heterogeneity	egger_intercept	p-value for pleiotropy
IVW	0.067		
MR Egger	0.069	0.003	0.302

MR, mendelian randomization; IVW, inverse variance weighted.

## 4 Discussion

In this study, we utilized an open-access GWAS dataset and bidirectional two-sample MR methods to comprehensively evaluate the relationship between OSA and TL. The results of forward MR analysis, using IVW, WM, and weighted mode MR methodologies, demonstrated a negative association between OSA and TL. This suggests a close association between OSA and telomere depletion. Although the MR-Egger findings were not statistically significant, possibly due to the method accommodating horizontal pleiotropy, resulting in wider confidence intervals and potential biases (Burgess and Thompson, 2017; Rees et al., 2017). This underscores the importance of considering methodological limitations when making causal inferences. On the other hand, reverse MR analysis substantiated the absence of a causal relationship between TL and OSA risk, indicating that a reduction in telomere length does not directly cause the onset of OSA. Sensitivity analyses and heterogeneity tests further affirmed the robustness of our findings. In summary, this study suggests that while OSA may expedite the depletion of TL, no causal association exists between TL and OSA risk.

Based on the literature search to date, this research is the inaugural study employing MR techniques to scrutinize the causal relationship between OSA and TL. Our results substantiated a negative causal linkage between OSA and TL, which aligns with previous research outcomes. For instance, a meta-analysis incorporating seven case-control studies along with one cohort study, involving a total of 2,639 participants, revealed that individuals with OSA have significantly shorter TL compared to their healthy counterparts (mean difference: −0.03; 95%CI: −0.06 to −0.00; P = 0.003) (Huang et al., 2018). Subgroup analyses based on age and sample size further reinforce this observation. Moreover, after accounting for demographic and lifestyle factors, cross-sectional studies demonstrated a significant association between severe OSA symptoms and reduced TL (P = 0.007) (Carroll et al., 2019). Additionally, a pilot study revealed that after 6 months of Continuous Positive Airway Pressure (CPAP) treatment, significant alleviation of hypoxia symptoms was observed in OSA patients, accompanied by an increase in TL (P = 0.03) (Madaeva et al., 2022).

While the mechanisms underlying the association between OSA and TL remain to be conclusively elucidated, extant research supports the hypothesis that oxidative stress and inflammation serve as foundational elements in establishing a negative causal relationship between the two (Kim et al., 2016; Turkiewicz et al., 2021). As such, this reinforces our findings that OSA serves as a risk factor for TL shortening. Specifically, the prevalent conditions of chronic intermittent hypoxia and sleep fragmentation in OSA patients disrupt the oxygen equilibrium in the bloodstream, thus precipitating oxidative stress. This sequence of events culminates in the production of a plethora of Reactive Oxygen Species (ROS), which inflict harm upon proteins, lipids, and DNA, thereby accelerating the shortening of TL (Cadet and Wagner, 2013). Moreover, heightened levels of various inflammatory indicators like Tumor Necrosis Factor-α, Interleukin-6, and C-Reactive Protein are frequently detected in the bloodstream of OSA patients, possibly triggering systemic inflammation and establishing a biological nexus between OSA and TL (Zhang et al., 2016). Concurrently, OSA is often accompanied by endocrine imbalances, notably fluctuations in cortisol levels, which may further modulate telomerase activity, thus indirectly affecting TL. Research by TempAku PF also suggested that OSA may affect telomerase activity by inhibiting the expression of KLOTHO protein, thereby connecting OSA and TL (Tempaku et al., 2021). TL serves as a key biomarker for biological aging and is connected to various age-related diseases, whereas OSA is intimately linked with a multitude of health challenges, including but not limited to cardiovascular disorders (Labarca et al., 2018; Huang et al., 2020; Ooi and Rajendran, 2023). Given that these phenomena may interact through complex biochemical mechanisms and genetic regulations, it becomes particularly crucial to gain a deeper understanding of the impact of OSA on biological aging. Consequently, our research sheds light on the potential negative causal relationship between OSA and TL, providing new perspectives for comprehending their interplay. This suggests that alleviating OSA symptoms may be significant for delaying cellular aging and maintaining telomere stability. Timely treatment of OSA may not only emerge as a vital strategy for combating aging but also afford novel insights for the prevention of age-related diseases.

The present study is characterized by multiple noteworthy strengths. First, we employed a two-sample MR design based on large-scale GWAS data, effectively minimizing the bias introduced by unobserved confounding variables and thus more accurately establishing the causal link between OSA and TL. Second, we conducted a bidirectional causality analysis to comprehensively ensure the exclusion of misleading causal effects when exploring the relationship between OSA and TL. Lastly, to holistically evaluate the causal effects, we used a variety of advanced statistical methods, including IVW, WM, Weighted Mode, and MR-Egger regression.

This study is subject to several limitations. The reliance on GWAS data from European populations limited the global applicability of our findings. Further MR studies involving diverse ethnic groups are warranted to corroborate these findings. Second, there may be differential effects on TL among OSA patients based on gender, age, and severity level. Unfortunately, the absence of stratified GWAS data precludes a more comprehensive analysis. Third, the IVs currently available for causal inference were relatively limited. However, as GWAS research evolves, we expect to identify a greater number of genetic markers strongly associated with OSA. To summarize, the study did shed light on the relationship between OSA and diminished TL. Nonetheless, the exact mechanisms contributing to this correlation warrant further in-depth investigation.

## 5 Conclusion

Overall, our study definitively demonstrated that OSA substantially hastens the deterioration of telomeres, which bears significant implications for clinical practice, particularly given that accelerated telomere degradation is linked to numerous ailments and shortened lifespan. Nevertheless, we found no causal relationship between TL and the risk of OSA onset. This insight offers a novel direction for subsequent studies, implying that TL may not be a dependable indicator for assessing the risk of OSA.

## Data Availability

The original contributions presented in the study are included in the article/Supplementary Material, further inquiries can be directed to the corresponding author.
