# Supplementary material for: Assessment of the causal association between obstructive sleep apnea and telomere length: a bidirectional mendelian randomization study
Source: Front Genet. 2025 Mar 4;16:1294105. doi: 10.3389/fgene.2025.1294105 (PMC11913802; doi:10.3389/fgene.2025.1294105)
Supplement: Supplementary file 1 [file DataSheet1.zip › Supplementary Material and Tables/Supplementary Material.DOCX]

Supplementary Material

Table S1:Basic information on instrumental variables(n=8) associated with OSA

| **SNP** | **EA** | **OA** | **EAF** | **β** | **SE** | **P value** | **F-statistic** |
| --- | --- | --- | --- | --- | --- | --- | --- |
| rs9937053 | A | G | 0.430 | 0.102 | 0.013 | 1.54E-07 | 66.586 |
| rs527014 | T | C | 0.074 | 0.125 | 0.024 | 2.80E-08 | 27.408 |
| rs4837016 | A | G | 0.466 | -0.071 | 0.013 | 2.91E-07 | 31.900 |
| rs3996329 | A | G | 0.237 | -0.076 | 0.015 | 1.53E-08 | 26.162 |
| rs193546 | A | G | 0.744 | 0.074 | 0.014 | 2.80E-11 | 26.707 |
| rs142006783 | C | T | 0.038 | 0.178 | 0.033 | 2.27E-07 | 29.731 |
| rs10928560 | T | C | 0.195 | -0.088 | 0.016 | 4.32E-16 | 30.880 |
| rs10507084 | T | C | 0.179 | 0.109 | 0.016 | 4.81E-08 | 44.308 |

 OSA, Obstructive Sleep Apnea; SNP, single nucleotide polymorphism; EA, effect allele; OA, Other allele; MAF, minor allele frequency; SE, standard error; β, causal effect coefficient.

Table S2: Basic information on instrumental variables(n=130) associated with TL

| **SNP** | **EA** | **OA** | **EAF** | **β** | **SE** | **P value** | **F-statistic** |
| --- | --- | --- | --- | --- | --- | --- | --- |
| rs1003322 | A | C | 0.214 | 0.014 | 0.002 | 1.00E-08 | 32.782 |
| rs10112752 | A | G | 0.430 | -0.029 | 0.002 | 9.50E-46 | 201.565 |
| rs1023767 | A | G | 0.238 | -0.018 | 0.002 | 5.00E-15 | 61.246 |
| rs10768683 | G | C | 0.841 | 0.047 | 0.003 | 1.50E-64 | 287.770 |
| rs10773176 | G | A | 0.741 | -0.017 | 0.002 | 5.20E-14 | 56.650 |
| rs10774624 | A | G | 0.533 | 0.015 | 0.002 | 2.90E-13 | 53.243 |
| rs10805346 | C | T | 0.439 | 0.012 | 0.002 | 7.00E-09 | 33.541 |
| rs10840270 | G | C | 0.656 | 0.014 | 0.002 | 1.30E-11 | 45.815 |
| rs10845387 | A | G | 0.353 | -0.014 | 0.002 | 1.50E-11 | 45.480 |
| rs10905255 | T | G | 0.579 | -0.018 | 0.002 | 2.60E-19 | 80.738 |
| rs11085072 | T | C | 0.237 | -0.013 | 0.002 | 2.60E-08 | 31.005 |
| rs11117354 | C | T | 0.697 | 0.023 | 0.002 | 3.40E-26 | 112.098 |
| rs111527438 | C | T | 0.351 | 0.013 | 0.002 | 3.10E-09 | 35.090 |
| rs111950327 | C | G | 0.064 | 0.024 | 0.004 | 5.90E-09 | 33.871 |
| rs112394943 | C | T | 0.163 | -0.020 | 0.003 | 1.60E-12 | 49.905 |
| rs113525195 | A | C | 0.290 | -0.012 | 0.002 | 3.10E-08 | 30.645 |
| rs11557154 | T | C | 0.130 | -0.034 | 0.003 | 1.10E-30 | 132.559 |
| rs11579626 | C | A | 0.085 | 0.027 | 0.004 | 1.30E-13 | 54.916 |
| rs11584821 | T | C | 0.176 | -0.031 | 0.003 | 3.00E-31 | 135.189 |
| rs116863223 | A | G | 0.012 | -0.082 | 0.009 | 2.60E-18 | 76.164 |
| rs11699829 | A | G | 0.034 | 0.064 | 0.006 | 1.50E-26 | 113.705 |
| rs117034449 | A | G | 0.023 | 0.037 | 0.007 | 2.10E-08 | 31.419 |
| rs117407747 | T | C | 0.028 | 0.045 | 0.006 | 1.80E-13 | 54.246 |
| rs117512405 | A | G | 0.017 | -0.079 | 0.008 | 9.50E-22 | 91.813 |
| rs117630647 | A | G | 0.021 | 0.060 | 0.007 | 1.40E-16 | 68.363 |
| rs11769630 | A | T | 0.072 | -0.026 | 0.004 | 4.30E-11 | 43.476 |
| rs11991877 | A | T | 0.889 | -0.030 | 0.003 | 3.20E-21 | 89.434 |
| rs12369950 | C | T | 0.141 | -0.018 | 0.003 | 8.00E-10 | 37.751 |
| rs12412214 | A | G | 0.280 | -0.025 | 0.002 | 3.40E-28 | 121.218 |
| rs12451892 | C | T | 0.381 | -0.012 | 0.002 | 2.20E-08 | 31.307 |
| rs1291143 | C | A | 0.849 | 0.049 | 0.003 | 1.80E-69 | 310.393 |
| rs12925933 | C | A | 0.662 | -0.015 | 0.002 | 7.00E-12 | 47.033 |
| rs12932179 | G | A | 0.561 | -0.014 | 0.002 | 1.80E-11 | 45.160 |
| rs13062095 | C | T | 0.328 | 0.014 | 0.002 | 9.70E-11 | 41.874 |
| rs13230646 | C | T | 0.249 | -0.017 | 0.002 | 8.90E-14 | 55.603 |
| rs1332941 | G | A | 0.820 | 0.026 | 0.003 | 5.90E-21 | 88.210 |
| rs137901416 | A | G | 0.100 | 0.046 | 0.003 | 4.70E-43 | 189.238 |
| rs139669835 | T | C | 0.009 | -0.061 | 0.011 | 6.10E-09 | 33.812 |
| rs139795227 | C | A | 0.014 | 0.060 | 0.009 | 6.70E-12 | 47.112 |
| rs142426306 | T | C | 0.040 | -0.050 | 0.005 | 8.70E-21 | 87.445 |
| rs143190905 | T | G | 0.080 | -0.072 | 0.004 | 1.60E-85 | 384.086 |
| rs144204502 | T | C | 0.013 | -0.101 | 0.009 | 3.40E-28 | 121.249 |
| rs145114957 | G | C | 0.043 | 0.027 | 0.005 | 4.60E-08 | 29.860 |
| rs150150565 | T | C | 0.021 | 0.064 | 0.007 | 6.80E-18 | 74.269 |
| rs16978028 | T | A | 0.144 | -0.030 | 0.003 | 8.20E-26 | 110.345 |
| rs17445108 | A | G | 0.127 | -0.017 | 0.003 | 2.00E-08 | 31.498 |
| rs17677991 | G | C | 0.342 | 0.022 | 0.002 | 4.40E-26 | 111.567 |
| rs17803849 | T | C | 0.405 | 0.027 | 0.002 | 4.20E-41 | 180.268 |
| rs182059586 | C | T | 0.025 | -0.057 | 0.007 | 4.90E-17 | 70.373 |
| rs185174247 | A | G | 0.056 | 0.037 | 0.004 | 1.10E-17 | 73.400 |
| rs188918174 | T | C | 0.036 | 0.040 | 0.005 | 1.20E-13 | 54.977 |
| rs1907702 | A | G | 0.767 | 0.015 | 0.002 | 5.90E-10 | 38.340 |
| rs1957937 | T | A | 0.160 | 0.021 | 0.003 | 1.90E-14 | 58.659 |
| rs1985369 | G | A | 0.868 | -0.031 | 0.003 | 3.60E-25 | 107.403 |
| rs2056726 | A | G | 0.214 | -0.023 | 0.002 | 7.90E-21 | 87.635 |
| rs2230590 | C | T | 0.511 | -0.016 | 0.002 | 3.60E-15 | 61.927 |
| rs2282764 | G | A | 0.142 | -0.022 | 0.003 | 9.30E-15 | 60.039 |
| rs2293579 | A | G | 0.386 | -0.013 | 0.002 | 3.30E-10 | 39.504 |
| rs2555104 | C | A | 0.434 | -0.014 | 0.002 | 6.60E-12 | 47.139 |
| rs2763979 | T | C | 0.360 | -0.028 | 0.002 | 1.30E-40 | 178.107 |
| rs28363070 | A | G | 0.013 | 0.076 | 0.010 | 3.50E-15 | 61.945 |
| rs28502153 | A | C | 0.378 | -0.022 | 0.002 | 1.20E-25 | 109.637 |
| rs28577594 | C | G | 0.710 | 0.019 | 0.002 | 5.40E-17 | 70.168 |
| rs2967355 | C | A | 0.774 | -0.046 | 0.002 | 4.00E-83 | 373.102 |
| rs2977608 | C | A | 0.744 | 0.013 | 0.002 | 3.00E-08 | 30.694 |
| rs3093888 | A | G | 0.051 | -0.029 | 0.005 | 1.50E-10 | 41.004 |
| rs35446936 | A | G | 0.244 | -0.094 | 0.002 | 1.00E-200 | 1628.819 |
| rs35640778 | A | G | 0.021 | -0.209 | 0.007 | 9.59E-195 | 886.250 |
| rs3767952 | A | G | 0.227 | 0.013 | 0.002 | 1.80E-08 | 31.703 |
| rs3785074 | G | A | 0.290 | 0.024 | 0.002 | 2.60E-27 | 117.168 |
| rs3891167 | G | A | 0.253 | -0.043 | 0.002 | 1.20E-70 | 315.777 |
| rs41269079 | A | T | 0.189 | 0.015 | 0.003 | 1.70E-09 | 36.294 |
| rs41304832 | A | G | 0.012 | 0.061 | 0.009 | 5.00E-11 | 43.174 |
| rs429358 | C | T | 0.154 | 0.017 | 0.003 | 3.80E-10 | 39.205 |
| rs4498805 | T | G | 0.547 | 0.015 | 0.002 | 5.70E-14 | 56.489 |
| rs4530278 | T | G | 0.598 | 0.014 | 0.002 | 1.50E-11 | 45.540 |
| rs45604339 | T | C | 0.342 | -0.020 | 0.002 | 4.30E-22 | 93.394 |
| rs4616688 | T | G | 0.525 | -0.017 | 0.002 | 4.50E-18 | 75.086 |
| rs4695407 | G | A | 0.508 | 0.014 | 0.002 | 1.50E-12 | 50.101 |
| rs4724 | A | G | 0.117 | -0.055 | 0.003 | 9.80E-69 | 307.006 |
| rs4731541 | G | C | 0.625 | -0.021 | 0.002 | 1.40E-23 | 100.153 |
| rs4743037 | T | C | 0.231 | 0.015 | 0.002 | 5.10E-10 | 38.624 |
| rs55747751 | A | G | 0.077 | -0.021 | 0.004 | 1.70E-08 | 31.815 |
| rs56799554 | G | A | 0.170 | -0.026 | 0.003 | 3.00E-22 | 94.069 |
| rs5742915 | C | T | 0.446 | 0.019 | 0.002 | 1.60E-21 | 90.846 |
| rs59409453 | G | A | 0.731 | 0.020 | 0.002 | 1.60E-18 | 77.118 |
| rs6007020 | C | T | 0.368 | 0.014 | 0.002 | 4.80E-12 | 47.778 |
| rs6054257 | A | G | 0.794 | -0.014 | 0.002 | 1.10E-08 | 32.711 |
| rs611646 | A | T | 0.409 | -0.037 | 0.002 | 3.50E-73 | 327.412 |
| rs61405042 | T | C | 0.029 | -0.050 | 0.006 | 8.50E-17 | 69.282 |
| rs61748181 | T | C | 0.029 | -0.059 | 0.006 | 2.80E-23 | 98.800 |
| rs6536702 | A | G | 0.775 | 0.053 | 0.002 | 9.40E-111 | 500.014 |
| rs6584579 | G | A | 0.399 | 0.011 | 0.002 | 2.00E-08 | 31.527 |
| rs6587577 | G | A | 0.826 | -0.018 | 0.003 | 4.80E-12 | 47.752 |
| rs6659669 | T | C | 0.605 | -0.012 | 0.002 | 1.10E-08 | 32.571 |
| rs6669563 | A | G | 0.438 | 0.018 | 0.002 | 2.10E-19 | 81.115 |
| rs66731853 | A | G | 0.317 | -0.018 | 0.002 | 1.50E-16 | 68.115 |
| rs6751209 | C | T | 0.204 | -0.014 | 0.002 | 1.60E-08 | 31.960 |
| rs6776756 | A | G | 0.598 | -0.017 | 0.002 | 1.10E-17 | 73.300 |
| rs6790988 | G | A | 0.742 | 0.015 | 0.002 | 1.80E-10 | 40.699 |
| rs6881568 | A | C | 0.363 | 0.017 | 0.002 | 3.70E-16 | 66.385 |
| rs7099229 | A | G | 0.273 | -0.015 | 0.002 | 8.40E-12 | 46.662 |
| rs7164950 | G | A | 0.406 | 0.013 | 0.002 | 2.30E-10 | 40.211 |
| rs7209057 | A | G | 0.561 | 0.012 | 0.002 | 5.70E-09 | 33.943 |
| rs7221585 | T | C | 0.224 | 0.014 | 0.002 | 6.70E-09 | 33.634 |
| rs73581419 | T | C | 0.107 | 0.023 | 0.003 | 1.30E-12 | 50.273 |
| rs73730598 | A | G | 0.055 | 0.027 | 0.004 | 4.70E-10 | 38.803 |
| rs75664430 | G | C | 0.248 | -0.024 | 0.002 | 3.60E-24 | 102.883 |
| rs76065543 | T | C | 0.138 | 0.034 | 0.003 | 4.20E-32 | 139.084 |
| rs76219171 | A | G | 0.058 | 0.036 | 0.004 | 7.80E-17 | 69.466 |
| rs762679 | A | T | 0.857 | 0.031 | 0.003 | 1.40E-27 | 118.373 |
| rs76666449 | C | T | 0.101 | 0.030 | 0.003 | 8.20E-19 | 78.458 |
| rs7705526 | A | C | 0.327 | 0.078 | 0.002 | 1.00E-200 | 1289.265 |
| rs77231040 | C | G | 0.006 | 0.099 | 0.013 | 2.00E-13 | 53.982 |
| rs7772289 | T | G | 0.503 | 0.018 | 0.002 | 1.70E-18 | 76.990 |
| rs77732866 | A | G | 0.138 | 0.018 | 0.003 | 9.20E-10 | 37.496 |
| rs7790856 | T | C | 0.289 | -0.044 | 0.002 | 1.80E-87 | 393.042 |
| rs78491606 | C | A | 0.018 | -0.076 | 0.007 | 1.90E-24 | 104.128 |
| rs80324517 | A | G | 0.048 | 0.040 | 0.005 | 1.80E-17 | 72.313 |
| rs8102497 | A | G | 0.432 | -0.015 | 0.002 | 1.40E-13 | 54.709 |
| rs8105767 | G | A | 0.295 | 0.033 | 0.002 | 2.50E-50 | 222.565 |
| rs869785 | C | T | 0.672 | -0.015 | 0.002 | 4.40E-12 | 47.915 |
| rs871134 | T | C | 0.569 | -0.018 | 0.002 | 1.70E-19 | 81.551 |
| rs932002 | T | C | 0.151 | -0.040 | 0.003 | 7.30E-47 | 206.672 |
| rs9398196 | G | A | 0.520 | -0.014 | 0.002 | 9.50E-13 | 50.942 |
| rs939916 | A | G | 0.670 | 0.024 | 0.002 | 6.60E-29 | 124.475 |
| rs9419958 | C | T | 0.861 | -0.081 | 0.003 | 2.60E-167 | 760.033 |
| rs9600019 | T | C | 0.336 | 0.013 | 0.002 | 2.40E-09 | 35.594 |
| rs9878436 | T | C | 0.434 | -0.014 | 0.002 | 1.20E-12 | 50.491 |
| rs9955360 | A | C | 0.869 | -0.019 | 0.003 | 2.20E-10 | 40.299 |

 TL, Telomere Length;  SNP, single nucleotide polymorphism; EA, effect allele; OA, Other allele; MAF, minor allele frequency; SE, standard error; β, causal effect coefficient.
