# Supplementary material for: Assessment of the causal association between obstructive sleep apnea and telomere length: a bidirectional mendelian randomization study
Source: Front Genet. 2025 Mar 4;16:1294105. doi: 10.3389/fgene.2025.1294105 (PMC11913802; doi:10.3389/fgene.2025.1294105)
Supplement: Supplementary file 1 [file DataSheet1.zip › Supplementary Material and Tables/Table 1.DOCX]

Table 1：Summary of the GWAS

| Trait | GWAS ID | Sample size | N SNPs | Population | PubMed ID |
| --- | --- | --- | --- | --- | --- |
| OSA | finn-b-G6_SLEEPAPNO | 217,955 | 16,380,465 | European | 33243845 |
| TL | ieu-b-4879 | 472,174 | 20,134,421 | European | 34611362 |

OSA, Obstructive Sleep Apnea; TL, Telomere Length; N SNPs, Numbers of single nucleotide polymorphisms.
