# Supplementary material for: Assessment of the causal association between obstructive sleep apnea and telomere length: a bidirectional mendelian randomization study
Source: Front Genet. 2025 Mar 4;16:1294105. doi: 10.3389/fgene.2025.1294105 (PMC11913802; doi:10.3389/fgene.2025.1294105)
Supplement: Supplementary file 1 [file DataSheet1.zip › Supplementary Material and Tables/Table 2.DOCX]

Table 2: Forward MR analysis of OSA on TL

| MR Methods | N SNPs | β | SE | OR（95%CI） | p-value |
| --- | --- | --- | --- | --- | --- |
| IVW | 8 | -0.037 | 0.013 | 0.964(0.939 to 0.989) | 0.006 |
| MR Egger | 8 | -0.087 | 0.068 | 0.916(0.802 to 1.050) | 0.246 |
| Weighted median | 8 | -0.047 | 0.015 | 0.954 (0.926 to 0.983) | 0.002 |
| Weighted mode | 8 | -0.050 | 0.019 | 0.951 (0.914 to 0.989) | 0.032 |

OSA, Obstructive Sleep Apnea; TL, Telomere Length; N SNPs, Numbers of single nucleotide polymorphisms; MR, Mendelian Randomization; SE, standard error; β, causal effect coefficient; OR, Odds Ratio; IVW, Inverse variance weighted.
