# Supplementary material for: Assessment of the causal association between obstructive sleep apnea and telomere length: a bidirectional mendelian randomization study
Source: Front Genet. 2025 Mar 4;16:1294105. doi: 10.3389/fgene.2025.1294105 (PMC11913802; doi:10.3389/fgene.2025.1294105)
Supplement: Supplementary file 1 [file DataSheet1.zip › Supplementary Material and Tables/Table 3.docx]

Table 3: Analysis of Heterogeneity and Pleiotropy in Forward MR

| MR Methods | p-value for Heterogeneity | egger_intercept | p-value for Pleiotropy |
| --- | --- | --- | --- |
| IVW | 0.178 |  |  |
| MR Egger | 0.158 | 0.005 | 0.475 |

MR, Mendelian Randomization; IVW, Inverse variance weighted
