# Supplementary material for: Assessment of the causal association between obstructive sleep apnea and telomere length: a bidirectional mendelian randomization study
Source: Front Genet. 2025 Mar 4;16:1294105. doi: 10.3389/fgene.2025.1294105 (PMC11913802; doi:10.3389/fgene.2025.1294105)
Supplement: Supplementary file 1 [file DataSheet1.zip › Supplementary Material and Tables/Table 4.DOCX]

Table 4 Reverse MR analysis of TL on OSA

| MR Methods | N SNPs | β | SE | OR（95%CI） | p-value |
| --- | --- | --- | --- | --- | --- |
| IVW | 130 | -0.036 | 0.053 | 0.965 (0.870 to 1.070) | 0.499 |
| MR Egger | 130 | -0.115 | 0.0891 | 0.890 (0.742 to 1.070) | 0.219 |
| Weighted median | 130 | -0.068 | 0.083 | 0.934 (0.797 to 1.095) | 0.412 |
| Weighted mode | 130 | -0.119 | 0.095 | 0.888 (0.743 to 1.061) | 0.211 |

OSA, Obstructive Sleep Apnea; TL, Telomere Length; N SNPs, Numbers of single nucleotide polymorphisms; MR, Mendelian Randomization; SE, standard error; β: causal effect coefficient; OR, Odds Ratio; IVW, Inverse variance weighted.
